# The Perspective of Therapeutic Antibody Marketing in Iran: Trend and Estimation by 2025

**DOI:** 10.1155/2021/5569590

**Published:** 2021-03-30

**Authors:** Monireh Golpour, Pouya Vatanpour, Mina Amini, Majid Saeedi, Nasim Hafezi, Alireza Rafiei

**Affiliations:** ^1^Molecular and Cell Biology Research Center, Student Research Committee, Faculty of Medicine, Mazandaran University of Medical Science, Sari, Iran; ^2^Oneocean Company, Oslo, Norway; ^3^Food and Drug Deputy, Mazandaran University of Medical Science, Sari, Iran; ^4^Departments of Pharmaceutics and Medicinal Chemistry, Faculty of Pharmacy, Mazandaran University of Medical Sciences, Sari, Iran; ^5^Department of Immunology, School of Medicine, Mazandaran University of Medical Sciences, Sari, Iran

## Abstract

**Background:**

Monoclonal antibodies with high efficiency and specificity are one of the best strategies to diagnose and treat a variety of diseases such as cancer, autoimmunity, and inflammatory diseases. The market for monoclonal therapeutic antibodies (MTAs) has grown dramatically in the past decade.

**Objective:**

Given the importance of these issues, developing countries spend a high cost on importing or producing MTAs annually. This study intends to examine the market of monoclonal therapeutic antibodies in Iran and predict the future growth rate of this market using the obtained data.

**Methods:**

Data on the status of MTAs in the country (from 2008 to 2018) were obtained from the Food and Drug Deputy of Mazandaran University of Medical Sciences. The market status of MTAs was studied based on the dosage forms, application, and price. Then, the market outlook was predicted up to year 2025.

**Results:**

The results showed that 58.8% of all MTAs were humanized, and 86% of all antibody-based drugs were used to treat cancer. Sales of MTA-based medications will reach $454 million by 2025 and are projected to grow significantly in the future.

**Conclusion:**

Given the increasing technology of the production of MTAs and their use in targeted therapies worldwide, their consumption market in Iran is expected to grow significantly.

## 1. Introduction

**Figure 1:**
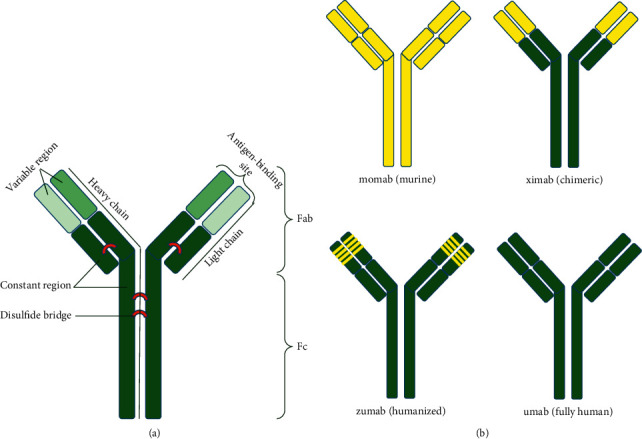
(a) A schematic representation of the antibody structure. (b) Various monoclonal antibodies based on different origins. Yellow parts show the murine-based source, and green parts show human-based sources.

Antibodies are glycoproteins that are produced by B lymphocytes in response to antigens, bind specifically to antigens, and play an important role in immune defense (Figure 1(a)) [1]. Antibodies can be classified into two types: polyclonal and monoclonal. Different clones of B cells produce polyclonal antibodies against different epitopes of an antigen. Monoclonal antibodies (mAbs) are produced by a B-cell clone against only one antigenic epitope. The exclusive features of mAbs, such as unique structure, high specificity, and relatively easy production, have increased the use of mAbs in the treatment of various diseases [2, 3]. In 1986, the first mAb, named OKT3 (muromonab), was approved and used in transplant recipients to prevent acute rejection [4, 5]. Subsequently, many antibodies have been developed to treat many diseases, such as cancer, autoimmune diseases, and inflammatory disorders [6]. The success of mAbs as targeted therapies has led to the emergence of an antibody industry whose application is growing. Thus, mAbs have been named as biological gold [5, 6].

mAbs are divided into four types according to their origin: murine mAbs, chimeric, humanized, and fully human (Figure 1(b)). Murine mAbs, with the suffix “-momab,” are constructed entirely of mice.

The most crucial drawback of this type of mAbs is the high immunogenicity, which leads to the production of human antimouse antibody (HAMA) responses that decrease its immunogenicity and efficacy. Because of these problems, other antibodies were designed that decrease the antihuman immunogenicity [7, 8]. Chimeric Abs are engineered human immunoglobulins with the extension of “-ximab,” in which the variable domain of the mouse substitutes their variable domain. This approach significantly reduces the production of HAMA responses compared to “murine mAbs,” but in some cases, such as Centicor/JNJ's Remicade (infliximab), human antichimeric antibody (HACA) responses have been observed that can lead to some problems and decrease mAb efficiency. Humanized mAbs with the suffix “-zumab” are designed so that only immunoglobulin complementary determining regions (CDRs) are derived from the mouse origin, and the rest of the molecule is derived from a human source [9–11]. Therefore, this strategy significantly leads to a decrease in the development of HAMA and HACA responses. To eliminate any immunogenicity, the fully human antibodies with the suffix “umab” are completely derived from a human source [10]. The key features of the new generation of antibodies are to minimize side effects and reduce therapeutic dose [7, 10, 11].

Today, thanks to the tremendous technological advances in the production of antibodies, our increased knowledge of the pathogenesis of many diseases, as well as the approach of physicians to personalized medicine, has led to the dramatic growth of the production of these MTA products [9]. Therefore, many countries spend millions of dollars every year to purchase MTA [4, 12]. According to a 2019 report, the approved mAbs' market reached 115.2 billion in 2018 and an increase of 11%, nearly $125 billion, in 2019 worldwide [13, 14], but the real-world market is beyond that and is projected to grow by 12.5 percent to $240 billion in 2025 [14].

The largest share (50%) of the MTA market belongs to North America, followed by Latin America, Western Europe, Eastern Europe, Asia-Pacific, the Middle East, Africa, and Japan [15]. Developing countries are the main consumer of mAbs in the world. They spend a lot of money annually importing or producing these products, and Iran is no exception.

On the contrary, an increase in the discovery and production of mAbs and an increase in our understanding of diseases at the molecular level, as well as a targeted treatment approach for many chronic diseases by physicians, have led to the increasing use of MTAs worldwide. This study intends to examine the market of monoclonal therapeutic antibodies in Iran and predict the future growth rate of this market using the obtained data. Accordingly, we first discussed the market situation over the last thirteen years with well-documented data and then predicted the market situation by 2025.

## 2. Materials and Methods

### 2.1. Data Extraction

Data on the status of MTAs in the country over the past thirteen years (2006 to 2018) are obtained from the Food and Drug Deputy of Mazandaran University of Medical Sciences. The data include the brand and generic name of the medication, dosage forms, amount or quantity required, and the price of the drug in US dollars. Since some of the arbitrators needed to be produced domestically, all prices were calculated in US dollars to equalize the cost of supplying MTA drugs.

### 2.2. Data Classification

The data were categorized by type and source, type of the original construct, clinical application, target molecule, and prices in US dollars. All the data were analyzed using Excel software.

### 2.3. Data Mining and Analysis

We used root-mean-square error (RMSE), mean absolute error (MAE), symmetric mean absolute percentage error (SMAPE), ease of movement (EMV), and R2 indexes (indicators) to analyze the annual use of MTAs and predict 5-year need. According to the results, the trend line has been calculated over the past thirteen years. Also, the EMV index examined the changes in the mean total costs and considered cost fluctuations (oscillations) based on useful indicators over the past thirteen years. Finally, an estimated model based on the MTA requirement by 2025 is calculated for our country.

## 3. Results

### 3.1. Frequency of Monoclonal Therapeutic Abs in the Past Thirteen Years

The distribution of MTAs uses from 2006 to 2018, as shown in Table 1. In total, 17 FDA-approved types of MTAs have been sold during the study period in Iran. They comprised 23.5% chimeric, 58.8% humanized, and 17.7% fully human mAbs. Based on clinical applications, 52.9% of them were prescribed for arthritis, inflammation, and immunity (AIID), 35.3% for oncology (ONCO), and the rest (11.8%) for both.

### 3.2. The Most Common MTAs in the Last Thirteen Years

**Figure 2:**
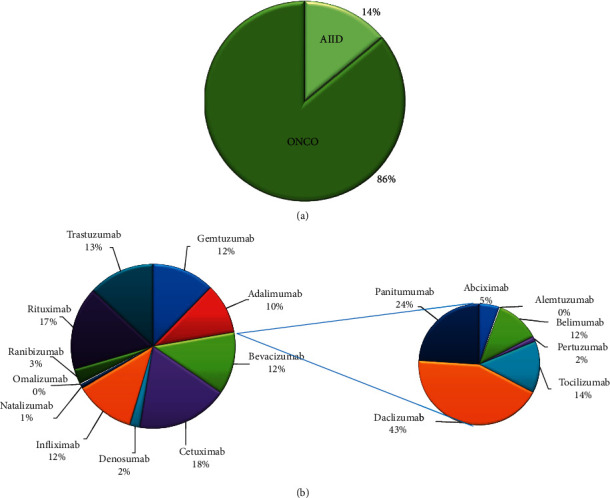
Monoclonal therapeutic antibodies (MTAs) in Iran from 2006 to 2018. The frequency of MTAs based on clinical application areas (a). The frequency of used MTAs by generic names (b).

The frequency of clinical applications of MTAs was calculated each year. Furthermore, from all, 86% were used in cancer and only about 14% in chronic inflammatory diseases (Figure 2(a)). In the next step, we categorized the usage frequency of the MTAs in each clinical area.  Figure 2(b) shows the overall frequency of each MTA used during the study period. The majority of them were commonly used for cancer treatment. Table 1 shows the target and clinical applications of MTAs that have been sold during the study period in Iran.

### 3.3. The Upward Trend in the Number and Market of MTAs over the Past Thirteen Years

**Figure 3:**
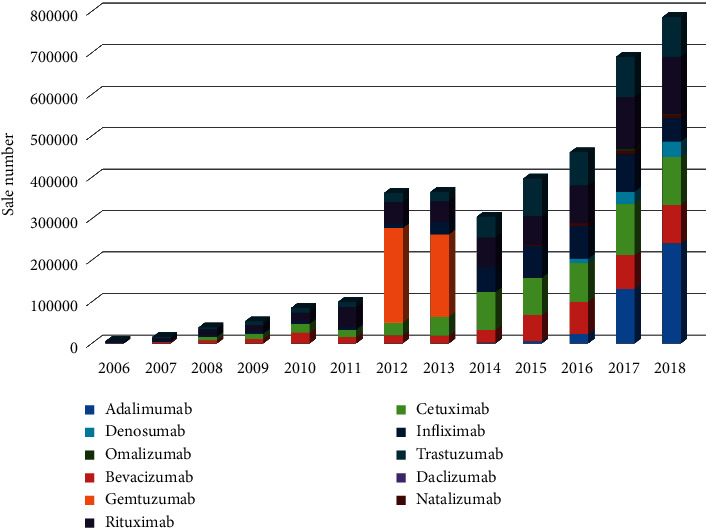
Frequency of MTAs used in Iran from 2006 to 2018.

As shown in Figure 3, the number and variety of MTAs increased dramatically from 2006 to 2018. In 2006, only four drugs, daclizumab, infliximab, rituximab, and trastuzumab, were used to treat cancer and chronic inflammatory diseases. Through the remarkable advances in biotechnology and immunology research over the last ten years, numerous antibodies have been discovered and produced around the world. In line with the increasing discovery and production of MTAs in the world, the MTA market in Iran has also grown exponentially. The most common MTAs in Iran consisted of infliximab (tumor necrosis factor (TNF)-a specific antibody), adalimumab (antitumor necrosis factor (TNF)-a specific antibody), trastuzumab (human epidermal growth factor receptor 2 (HER2)-a specific antibody), bevacizumab (vascular endothelial growth factor A (VEGF-A)-a specific antibody), and rituximab (anti-CD20-specific-antibody) (Figure 3). Among them, trastuzumab and rituximab were the frequently used MTAs in each study year.

**Figure 4:**
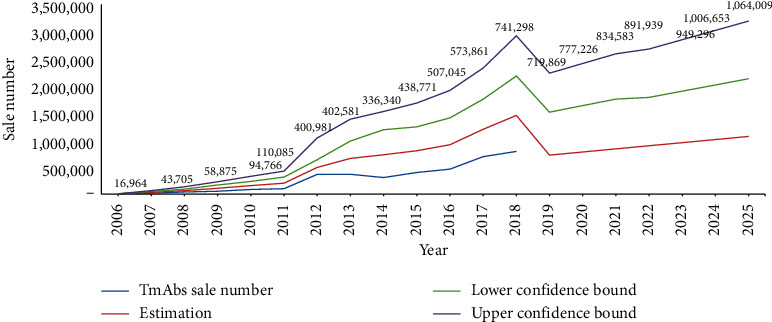
Sales of MTA items in Iran (2006–2018) and estimation to 2025.

Figure 4 shows a significant increase in the MTA market in Iran from 2006 to 2018. There was an upward trend from 5965 to 741,295 medicines in 2006 and 2018, indicating a 124.3-fold increase in this market for thirteen years (Figure 4). We based this incremental process and its related changes on a model for estimating the needs of these drugs by 2025. As shown in Figure 4, the demand for MTAs will reach more than 1,000,000 items in 2025.

### 3.4. A Predictive Model of the Cost of Importing MTAs over the Coming Years

**Figure 5:**
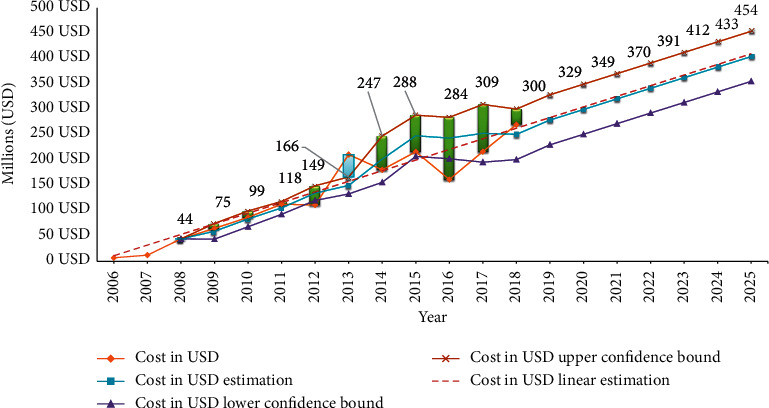
The cost of MTAs in Iran (2006–2018) and estimation to 2025.

Given the high consumption of MTAs in the last thirteen years and an increasing trend over the previous years, a consumption estimating model was presented in the coming years. As shown in Figure 5, the cost of providing these drugs will increase dramatically, with prices rising from $300 million in 2018 to over $454 million in 2025.

## 4. Discussion

Monoclonal antibodies with high efficiency, specificity, and less toxic effects are targeted tools for the development of personalized medicine for the diagnosis and treatment of various diseases, including cancer, inflammatory diseases, autoimmune disorders, and infectious diseases. More than hundreds of MTAs have been developed for over 30 years, and some have been approved. Although the MTA market has grown dramatically in the world, the major suppliers are the United States, Europe, Latin America, Asia, and Japan. In this study, we have evaluated the MTA market in Iran over the past thirteen years based on the therapeutic area and dosage form to provide a predictive model by 2025. The results showed that the source of all MTAs was 23.5% chimeric, 58.8% humanized, and 17.7% fully human. These data are consistent with the overall development of the MTA over time. As in the early years (2001 and 2002), the major (70%) MTAs were chimeric antibodies.

However, due to the immunogenicity problems of chimeric antibodies, the contribution of these types of antibodies in the global MTA market has decreased significantly. Instead, humanized and fully human MTAs rapidly replaced chimeric antibodies in the treatment of many disorders due to their low immunogenicity. Adalimumab was the first humanized antibody approved by the FDA in 2003 [2]. The benefits of genetic engineering, yeast or phage display technology, and transgenic mice have led to rapid growth in the production and clinical use of humanized monoclonal antibodies [16]. It, therefore, indicates that humanized antibodies play a significant role in predicting future needs' assessment. This may be either due to the more beneficial effects of the antibodies, especially immune checkpoint inhibitors, or due to the side effects of chemotherapy drugs that have caused MTAs to become more prominent. Our data showed that 68% of MTAs were used in cancer, and only 14% were used in chronic inflammatory diseases. The most common MTAs were cetuximab, rituximab, trastuzumab, gemtuzumab, bevacizumab, and infliximab, respectively, and denosumab, ranibizumab, and adalimumab antibodies accounted for only 15% of the MTA market in Iran. These antibodies are used to treat AML, CLL, non-Hodgkin's lymphoma, head and neck cancer, colorectal cancer, breast cancer, gastric/gastroesophageal carcinoma, nonsquamous non-small-cell lung cancer, renal cell carcinoma, glioblastoma, Crohn's disease, ulcerative colitis, RA, ankylosing spondylitis, and psoriatic arthritis [17–21].

Over the past two decades, monoclonal antibodies designed to target tumor cells or improve immune system exhaustion with fewer side effects and high efficacy have rapidly increased due to increased understanding of genomic studies and technological advances [22–24]. It is worth noting that, in recent years, more than 33 new therapeutic antibodies are in the late stage of the clinical phase to evaluate their therapeutic effect on cancer [25]. An important factor leading to the growth of anticancer MTAs is increasing cancer incidence and mortality worldwide [26]. Conventional cancer treatments include chemotherapy, radiotherapy, and surgery that have common side effects such as fever, infection, heart failure, and allergic reactions [3, 22, 23]. Moreover, high efficacy and fewer side effects are another reason why MTAs are more favored [27]. At this growth rate, we expect MTA sales in Iran to grow to $250 million by 2020 and close to $339 million by 2025, with the number of these drugs required to reach more than 10,000,000 annually. While the market for MTAs is currently estimated at $9.72 billion in 2018, estimation indicates that it will stabilize in North America by the end of 2026 to reach $26.16 billion [28].

## 5. Conclusion

Taken together, given the increasing technology of the production of these drugs and their use in targeted therapies worldwide, their consumption market in Iran is expected to grow significantly. Given the high cost of MTAs and the increasing need to use them in the near future, there should be an opportunity to improve investment in the research and development of MTA production technology in Iran to meet both domestic demand and export opportunity.

## Figures and Tables

**Table 1:** Characterization of monoclonal therapeutic antibodies (MTAs) used from 2007 to 2018 in Iran.

Generic name	Isotype	Target	Indications	Therapy field	FDA approval date	Source
Gemtuzumab	IgG4	CD33	Acute myeloid leukemia	ONCO	2000	Humanized

Cetuximab	IgG1K	EGFR	Head and neck cancer, KRAS-ve colorectal cancer	ONCO	2012	Chimeric

Inﬂiximab	IgG1K	TNF-*α*	Crohn's disease, ulcerative colitis, RA, ankylosing spondylitis, psoriatic arthritis	AIID	1998	Chimeric

Trastuzumab	IgG1K	HER2	Breast cancer, gastric/gastroesophageal carcinoma	ONCO	1998	Humanized

Bevacizumab	IgG1K	VEGF-A	Colorectal cancer, nonsquamous non-small-cell lung cancer, renal cell carcinoma, glioblastoma	ONCO	2004	Humanized

Rituximab	IgG1K	CD20	Non-Hodgkin's lymphoma, chronic lymphocytic leukemia, rheumatoid arthritis	ONCO, AIID	1997	Chimeric

Adalimumab	IgG1K	TNF-*α*	Rheumatoid arthritis, juvenile idiopathic arthritis, psoriatic arthritis, ankylosing spondylitis, Crohn's disease, plaque psoriasis	AIID	2002	Fully human

Natalizumab	IgG4K	Alpha-4 integrin	Multiple sclerosis, Crohn's disease	AIID	2004	Humanized

Daclizumab	IgG1	CD25	Acute transplant rejection, multiple sclerosis	AIID	1997	Humanized

Denosumab	IgG2	RANK ligand	Bone metastases from solid tumors, osteoporosis, giant cell tumor of the bone	ONCO	2010	Fully human

Belimumab	IgG1*λ*	BAFF, BLYs	Systemic lupus erythematosus	AIID	2011	Fully human

Tocilizumab	IgG1k	IL-6R	Rheumatoid arthritis	AIID	2010	Humanized

Ranibizumab	IgG1k	VEGF-A	Age-related macular degeneration, age-related vision loss	AIID	2015	Humanized

Abciximab	IgG1	CD41	Acute myocardial infarction	AIID	1993	Chimeric

Pertuzumab	IgG1k	HER2	Metastatic HER2-positive breast cancer, neoadjuvant in early HER2-positive breast cancer	ONCO	2012	Humanized

Omalizumab	IgG1k	IgE FC region	Severe allergic asthma	AIID	2014	Humanized

Alemtuzumab	IgG1k	CD52	Chronic lymphocytic leukemia (CLL), cutaneous T-cell lymphoma (CTCL), multiple sclerosis	ONCO, AIID	2013	Humanized

ONCO: oncology; AIID: arthritis, inflammation, and immune disorder.

## Data Availability

No data were used to support this study.
